# Genome sequences of 12 isolates of the EU1 lineage of *Phytophthora ramorum*, a fungus-like pathogen that causes extensive damage and mortality to a wide range of trees and other plants

**DOI:** 10.1016/j.gdata.2017.02.006

**Published:** 2017-02-06

**Authors:** Judith Turner, Paul O'Neill, Murray Grant, Rick A. Mumford, Richard Thwaites, David J. Studholme

**Affiliations:** a Fera Science Ltd (Fera), National Agri-Food Innovation Campus, Sand Hutton, York YO41 1LZ, United Kingdom; b Biosciences, University of Exeter, Stocker Road, Exeter EX4 4QD, United Kingdom

## Abstract

Here we present genome sequences for twelve isolates of the invasive pathogen *Phytophthora ramorum* EU1. The assembled genome sequences and raw sequence data are available *via* BioProject accession number PRJNA177509. These data will be useful in developing molecular tools for specific detection and identification of this pathogen.

**Table:** 

Specifications [standardized info for the reader]	
Organism/cell line/tissue	Twelve isolates of the EU2 lineage of *Phytophthora ramorum*
Sex	Not applicable
Sequencer or array type	Illumina HiSeq 2000 and Illumina GA II
Data format	Analysed; *i*.*e*. raw data filtered and assembled
Experimental factors	Genomic sequences of pure microbial cultures
Experimental features	Genomic sequences of pure microbial cultures
Consent	Not applicable; data are available without restriction
Sample source location	All isolates were collected in the United Kingdom

## 1 Direct link to deposited data

https://www.ncbi.nlm.nih.gov/bioproject/177509

## 2 Experimental design, materials and methods

Fungus-like pathogens belonging to the oomycete genus *Phytophthora* pose significant threats to a wide range of plants [1]. Recent studies have generated whole-genome sequence data for *Phytophthora* species that cause disease in trees [2], [3], [4], [5], [6]. *Phytophthora ramorum* is an exotic pathogen whose geographical origin is unknown. In North America, *P*. *ramorum* is responsible for Sudden Oak Death while in Europe it causes Sudden Larch Death and Ramorum Blight [7], [8], [9], [10]. Four distinct lineages are known, which have been isolated from each other for hundreds of millennia [11], [12], [13]. For more than a decade, a reference genome sequence was available [14] for NA1, the lineage that has established itself in the wild (*i*.*e*. outside of the nursery trade) in North America. No genome sequence was available for lineage EU1, the first lineage to be discovered in Europe and which has subsequently been detected in North America [15] [16].

We previously reported genome sequences [3] for one of the two lineages found in Europe, namely EU2. Here we present the first genome sequences for lineage EU1 isolates, which were collected from several host species in several counties of England (see Table 1). The availability of genome sequences from multiple lineages will help to address the question of what are the genetic differences that underlie observed phenotypic differences [17] among the lineages as well evolutionary relationships among lineages and the possibility of identifying lineage-specific molecular markers. Availability of sequence data from multiple isolates within a single lineage may further offer insights into the recent evolutionary events following colonization of a new geographical range and new host populations [18]. In the absence of sexual recombination in these diploid pathogens, one mechanism for rapid adaptation may be aneuploidy and/or loss of heterozygosity (LOH) [19], [20], [21], [22], [23].

**Table 1:** Isolates and raw sequence data. All samples were collected by The Plant Health and Seeds Inspectorate except for CC12475 and CC14654, which were collected by Fera.

Isolate	Year	Source	County	BioSample	SRA	Read 1 (bp)	Read 2 (bp)	Read pairs	Platform
CC12475	2007	Soil	Cornwall	SAMN01797768	SRX202256	73	73	10,929,957	HiSeq 2000
CC14654	2009	Leaf detritus	Cornwall	SAMN01797770	SRX202259	73	73	32,069,494	HiSeq 2000
CC2184	2004	*Taxus* sp.	Cornwall	SAMN01797769	SRX202257	73	73	26,720,144	HiSeq 2000
CC1008	2002	*Rhododendron* sp.	West Sussex	SAMN05823577	SRX2190141	100	80	35,377,462	HiSeq 2000
CC1033	2002	*Viburnum* sp.	Dorset	SAMN05823579	SRX2190142	100	80	29,037,290	HiSeq 2000
CC1048	2002	*Viburnum* sp.	Gloucestershire	SAMN01797771	SRX202261	73	73	19,206,505	HiSeq 2000
CC2168	2009	*Camellia* sp.	Cornwall	SAMN05823583	SRX2190143	100	80	31,267,535	HiSeq 2000
CC2176	2009	*Pieris* sp.	Cornwall	SAMN05823584	SRX2190144	100	80	32,285,291	HiSeq 2000
CC2186	2009	*Rhododendron* sp.	Devon	SAMN05823586	SRX2190145	100	80	22,816,927	HiSeq 2000
CC2187	2009	*Rhododendron* sp.	Cornwall	SAMN05823587	SRX2190146	100	80	28,439,345	HiSeq 2000
CC2275	2004	*Laurus nobilis*	Cornwall	SAMN01797766	SRX202258	100	100	94,913,722	GA IIx
CC1011	2002	*Rhododendron* sp.	Cheshire	SAMN01797767	SRX202260	100	100	191,582,259	GA IIx

Paired-reads were generated from genomic sequence libraries, following the manufacturer's instructions, on the Illumina HiSeq 2000 or Illumina GA IIx massively parallel sequencing platforms. Numbers of reads, lengths and database accession numbers for the raw reads are listed in Table 1.

We filtered low-quality data and contaminating adaptor sequences using TrimGalore [24], which wraps the Cutadapt tool [25]. The full TrimGalore command line was “trim_galore –q 30 –paired read1.fq read2.fq”. We then assembled the filtered reads using SPAdes 3.9.0 [26] with the following command line: “spades.py --careful -t 8 --pe1-1 read1-filtered.fq --pe1-2 read1-filtered.fq -o output-directory”. During submission of the assemblies to GenBank [27], we removed sequences identified by the NCBI curators as contamination from vectors, mitochondria, bacteria *etc*. Assembly statistics are summarized in Table 2.

**Table 2:** Assembly statistics.

Isolate	GenBank accession number	Total length (bp)	Genomic coverage	Number of contigs	Number of scaffolds	Contig N_50_ (bp)	Scaffold N_50_ (bp)
CC1008	MLJA00000000	39,143,618	53.4 ×	5099	4625	20,824	24,587
CC1033	MLJB00000000	39,265,443	41.4 ×	5112	4666	21,410	25,459
CC1048	MLJC00000000	39,184,277	28.3 ×	4887	4562	22,104	25,016
CC12475	MLIX00000000	38,872,797	17.0 ×	4749	4449	22,493	24,333
CC14654	MLIY00000000	38,990,779	47.0 ×	5403	4802	18,355	23,539
CC2168	MLJD00000000	39,189,198	47.6 ×	5208	4707	19,710	24,940
CC2176	MLJE00000000	39,297,625	46.6 ×	5050	4635	20,983	24,953
CC2184	MLIZ00000000	39,039,515	39.6 ×	5237	4706	19,158	24,300
CC2186	MLJF00000000	40,428,501	32.9 ×	6372	5378	19,249	23,332
CC2187	MLJG00000000	39,213,677	42.0 ×	5054	4612	21,714	25,338
CC2275	AMZZ00000000	38,865,974	162.0 ×	5507	2445	17,358	23,300
CC1011	MRWH00000000	40,766,767	310.2 ×	6920	5959	15,332	21,758

We assessed the completeness of the genome assemblies using BUSCO (Benchmarking Universal Single-Copy Orthologs) [28], which checks for the presence of single-copy orthologous genes commonly conserved across eukaryotes. BUSCO denotes each gene as “complete single copy”, “complete duplicated”, “fragmented”, or “missing” in the assembly. Table 2 shows the percentage of these 429 genes that are “complete single copy” in each genome assembly. The levels of completeness (83.22 to 84.15) are comparable to those of six recently published *Phytophthora* genomes [5], which had up to 82.8% completeness, as assessed by the same method (Table 3).

**Table 3:** Completeness of assemblies assessed using BUSCO [28].

Assembly	Complete single copy	Complete duplicated	Fragmented	Missing	Total
EU1 CC2168 (this study)	361 (84.15%)	58	9	59	429
EU1 CC2184 (this study)	361 (84.15%)	62	10	58	429
EU1 CC2187 (this study)	361 (84.15%)	62	10	58	429
EU2 SOD158 [3]	361 (84.15%)	62	11	57	429
EU1 CC2176 (this study)	360 (83.92%)	62	10	59	429
EU2 SOD136 [3]	360 (83.92%)	61	11	58	429
EU1 CC14654 (this study)	359 (83.68%)	58	11	59	429
EU2 996/3 (6)	359 (83.68%)	78	13	57	429
EU2 SOD22 [3]	359 (83.68%)	64	12	58	429
EU1 CC1008 (this study)	358 (83.45%)	62	12	59	429
EU1 CC1033 (this study)	358 (83.45%)	60	12	59	429
EU1 CC2186 (this study)	358 (83.45%)	66	11	60	429
EU2 SOD58 [3]	358 (83.45%)	59	13	58	429
EU1 CC12475 (this study)	357 (83.22%)	62	12	60	429
EU1 CC2275 (this study)	357 (83.22%)	56	12	60	429
EU2 SOD69 [3]	357 (83.22%)	61	14	58	429
EU2 SODL51 [3]	357 (83.22%)	64	13	59	429
EU1 CC1048 (This study)	356 (82.98%)	57	12	61	429
NA1 Pr102 [14]	351 (81.82%)	65	16	62	429

Average nucleotide identities (ANI) were calculated, using the *dnadiff* tool in MUMMer [29], [30], between EU1 and previously published assemblies of closely related genomes [3], [4], [5], [6], [14]. The *Pr* EU1 assembly shared 99.2% ANI with *Pr* NA1 and 98.7% ANI with *Pr* EU2 suggesting a more ancient divergence between EU1 and EU2 than between EU1 and NA1. Between *Pr* EU1 and its sister species *P*. *lateralis*, there was 91.5% ANI. The *dnadiff* analysis also revealed that 1.5% of the EU1 genome is not alignable against the previously published genomes of EU2 and NA1, suggesting that there is a significant complement of lineage-specific genome content, including genes encoding effector proteins.

**Fig. 1:**
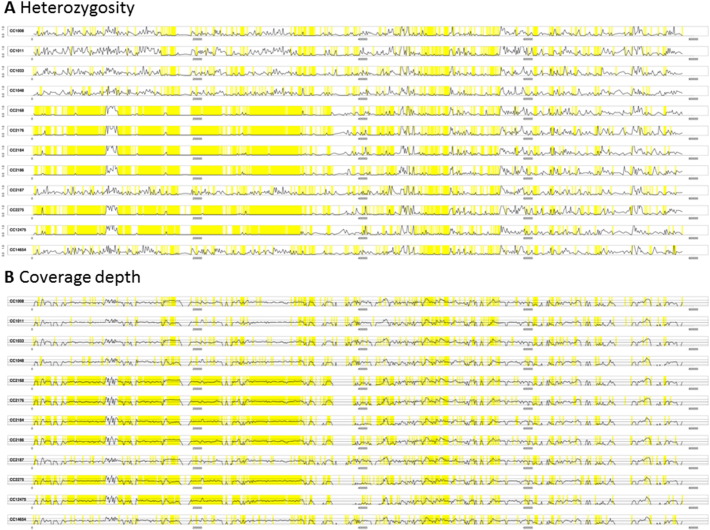
Heterozygosity profiles of twelve *Phytophthora ramorum* EU1 isolates over scaffold 7. The previously published *P*. *ramorum* NA1 genome sequence [14] was downloaded from the Joint Genome Institute at http://genome.jgi.doe.gov/ramorum1/ramorum1.download.ftp.html and used as a reference sequence, against which genomic sequence reads from each of the 12 isolates were aligned with BWA-mem [31], [32]. Panel A: we used a sliding window of 1000 nucleotides to calculate the rate of heterozygosity. Proportion of single-nucleotide positions at which 45–55% of the aligned reads contain the second-most abundant nucleotide was expressed as a percentage; that is the vertical axis represents percentage heterozygosity. Panel B: we used a sliding window of 1000 nucleotides to calculate average depth of coverage by aligned reads. The vertical axis represents depth of coverage, normalized so that the median depth over the whole genome is one. In both panels, the horizontal axis represents position on the scaffold and regions of zero heterozygosity are highlighted in yellow.

**Fig. 2:**
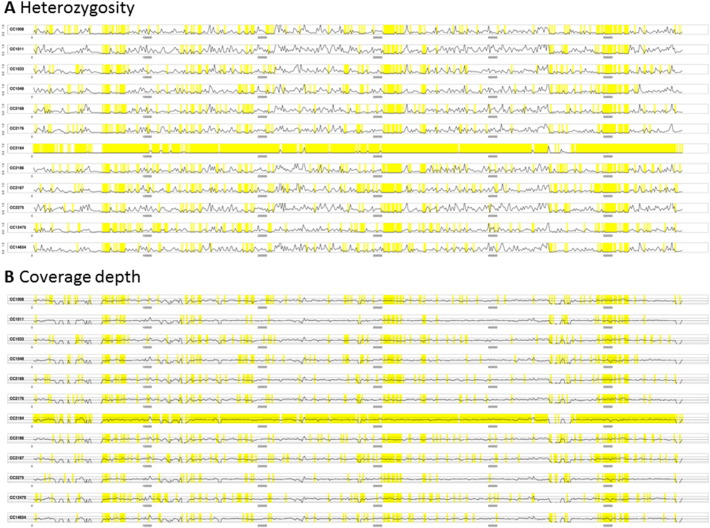
Heterozygosity profiles of twelve *Phytophthora ramorum* EU1 isolates over scaffold 24. The previously published *P*. *ramorum* NA1 genome sequence [14] was downloaded from the Joint Genome Institute at http://genome.jgi.doe.gov/ramorum1/ramorum1.download.ftp.html and used as a reference sequence, against which genomic sequence reads from each of the 12 isolates were aligned with BWA-mem [31], [32]. Panel A: we used a sliding window of 1000 nucleotides to calculate the rate of heterozygosity. Proportion of single-nucleotide positions at which 45–55% of the aligned reads contain the second-most abundant nucleotide was expressed as a percentage; that is the vertical axis represents percentage heterozygosity. Panel B: we used a sliding window of 1000 nucleotides to calculate average depth of coverage by aligned reads. The vertical axis represents depth of coverage, normalized so that the median depth over the whole genome is one. In both panels, the horizontal axis represents position on the scaffold and regions of zero heterozygosity are highlighted in yellow.

Heterozygosity has previously been observed in *P*. *ramorum* lineage NA1 [14] and is apparent in the newly presented data here for lineage EU1. We surveyed the distribution of heterozygosity across the genome by aligning sequence reads against the previously published genome sequence assembly of NA1 [14], which we downloaded from the Joint Genome Institute at http://genome.jgi.doe.gov/ramorum1/ramorum1.download.ftp.html. Prior to alignment using BWA-mem [31], [32], the reads were first filtered using TrimGalore as described above. The resulting alignment was converted to mpileup format using SAMtools [33]. By parsing the mpileup file, it was possible to count the number of sites that were probably homozygous (> 95% consensus among aligned reads) and those that were probably heterozygous (> 45% and < 55% consensus). Fig. 1 and Fig. 2 show plots of rates of heterozygosity respectively over scaffold 7 and scaffold 24 of the reference genome. On scaffold 7, there are large stretches with little or no heterozygosity in isolates CC2168, CC2176, CC2184, CC2186, CC2275 and CC12475 while the same regions show normal levels of heterozygosity in the other isolates. This suggests that CC2168, CC2176, CC2184, CC2186, CC2275 and CC12475 have undergone LOH in these regions of scaffold 7. The depths of sequencing coverage are normal (see panel B in Fig. 1) across the LOH regions, indicating that this is copy-number-neutral LOH rather than hemizygosity. Similarly, isolate CC2184 appears to have undergone copy-number-neutral LOH on scaffold 24 (Fig. 2); similar patterns can be observed on several other genomic scaffolds including scaffolds 11, 14, 16 and 33. It is not clear whether these putative LOH events occurred during growth on the host plant or whether they occurred subsequently in the laboratory after collection. However, a recent study of phenotypic and genetic variation in lineage NA1 concluded that partial aneuploidy and copy-neutral LOH were induced by the host. The most unique pattern of LOH among the EU1 isolates was observed for isolate CC2184 from yew (*Taxus* sp.); it would be interesting to survey additional isolates from this host and check whether they display the same distinctive LOH profile across their genomes.

Whole-genome sequence data are now available for multiple isolates of both of the *P*. *ramorum* lineages found in Europe, that is EU1 (this study) and EU2 [3], [6] as well as for the NA1 lineage found in North America [14]. As well as being a resource for biological and evolutionary research on this important invasive species, it also allows the identification of genomic sequences that could be targeted in new molecular tools for detection and identification of the species and lineages. Furthermore, identification of loci that are polymorphic among different isolates within the single lineage offers opportunities to track the spread of the pathogen in time and space at high resolution.
